# Oral diadochokinesis performance correlates with mild cognitive impairment: a cross-sectional study

**DOI:** 10.1186/s12903-025-06289-4

**Published:** 2025-06-03

**Authors:** Yuichi Ishihara, Akira Taguchi, Shiho Yunoue, Misaki Mugiyama, Kazumi Hosokubo, Minami Ido, Kaori Nohara, Momo Shinmei, Takayuki Oniki, Akira Uchiyama, Masae Furukawa, Jingshu Wang, Naoki Saji, Akinori Takeda, Takashi Sakurai, Kenji Matsushita

**Affiliations:** 1 Lion Foundation for Dental Health, Tokyo, Japan; 2 Department of Oral and Maxillofacial Radiology School of Dentistry, Matsumoto Dental University, Nagano, Japan; 3 Department of Oral Disease Research, National Center for Geriatrics and Gerontology, Geroscience Research Center, Research Institute, Aichi, Japan; 4 Center for Comprehensive Care and Research On Memory Disorders, Hospital, National Center for Geriatrics and Gerontology, Aichi, Japan; 5 Department of Dental Hygiene, Ogaki Women’s College, Gifu, Japan

**Keywords:** Cognitive dysfunction, Deglutition disorders, Dementia, Logistic models, Mild cognitive impairment

## Abstract

**Background:**

Oral dysfunctions that affect masticatory function, such as tooth loss, reportedly lead to malnutrition, which contributes to cognitive decline and the onset and progression of dementia. Previous studies on oral dysfunction have focused on older people already in need of nursing care. Therefore, we conducted a study of older people able to travel independently or with minor assistance to examine the relationship between oral and cognitive function in memory clinic study participants classified as cognitively normal, with mild cognitive impairment, or with dementia.

**Methods:**

Participants were 178 study participants (median age: 79.0 years, 49.4% female) attending the memory clinic at the National Center for Geriatrics and Gerontology, Japan. Following provision of informed consent, cognitive function was assessed using clinical dementia ratings and oral function assessed using number of present teeth, occlusal force, oral diadochokinesis, repetitive salivary swallowing test, and tongue pressure. The relationships between cognitive and oral function were evaluated using multivariable logistic regression analyses.

**Results:**

Of the participants, 25, 92, and 61 were classified as cognitively normal, with mild cognitive impairment, and with dementia, respectively. Oral diadochokinesis /ka/ was associated with a high risk of mild cognitive impairment: adjusted odds ratio, 6.930 (95% confidence interval: 1.284–37.402, *P* = 0.024). Repetitive salivary swallowing test score was associated with a high-risk tendency for dementia: adjusted odds ratio, 4.171 (95% confidence interval: 0.981–17.736, *P* for trend = 0.053). Number of present teeth, occlusal force, and tongue pressure were not associated with mild cognitive impairment or dementia.

**Conclusion:**

Oral diadochokinesis /ka/ was independently associated with mild cognitive impairment. A well-designed cohort study is needed to clarify the causal relationships between cognitive decline and oral diadochokinesis. The ability to identify study participants with possible mild cognitive impairment through regular dental examinations would help to prevent dementia.

**Trial registration:**

This study was registered to UMIN Clinical Trials Registry (UMIN000048126) at 2022–06-21.

**Supplementary Information:**

The online version contains supplementary material available at 10.1186/s12903-025-06289-4.

## Background

The global estimate for the number of people with dementia was 46.8 million people in 2015; this figure is expected to double every 20 years to reach 74.7 million in 2030, with particularly high prevalence in low-income countries in East Asia and Africa [1]. In higher-income countries, such as the United States and those in Europe, measures have been implemented to reduce the risk of developing dementia [2] by reducing factors such as hypertension [3], smoking [4], and diabetes [5, 6]. Therefore, the number of dementia study participants and dementia-related costs are expected to decline in such countries, highlighting the importance of identifying the risks associated with the development of dementia.

Several prospective cohort studies have shown that poor mastication is a risk factor for rapid decline in cognitive function and increased incidence of dementia [7]. One study showed that individuals with severe cognitive impairment had fewer occluding pairs and a smaller active mouth opening [8]. Examining the relationship between cognitive decline and oral function may be particularly useful for dementia prevention. Question-based screening tests, such as the Mini-Mental State Examination (MMSE) are often used to classify participants. However, it should be noted that although the MMSE can detect study participants with dementia, it is somewhat poor at detecting mild cognitive decline [9]. The Clinical Dementia Rating (CDR) scale is used to evaluate the severity of dementia, and classifies study participants according to five levels ranging from “cognitively normal” to “mild cognitive impairment (MCI)” and “severe dementia” [10]. Meta-analysis of the diagnostic accuracy of the CDR indicates that it is useful in detecting MCI and dementia compared with the MMSE [11]. MCI is an intermediate stage between normal aging and dementia. MCI study participants do not meet the clinical diagnostic criteria for Alzheimer’s disease, and tend to have age-related memory impairment but little or no problems with daily living [12]. The symptoms of mild dementia do not tend to improve; however, previous studies have shown that people with MCI may return to normal functioning or progress to dementia [13–15].

Recently, we reported that cognitive decline was independently and strongly associated with the presence of periodontal disease [16]. However, the relationships between oral function and MCI remain to be fully clarified. A greater understanding of the oral-related characteristics of MCI would facilitate the provision of professional dental care or oral functional training, which may be effective in preventing the onset of dementia.

Thus, we aimed to examine the relationship between oral and cognitive function in study participants attending a memory clinic and with CDR classifications of Cognitively normal, MCI, or dementia.

## Methods

### Study design

In this single-center observational study, which was named the “Clinical survey to clarify the association between periodontal disease, oral and cognitive functions: an observational study” (Pearl study), we aimed to investigate periodontal pathology and oral and cognitive functions in memory clinic outpatients at the National Center for Geriatrics and Gerontology (NCGG). The Pearl study was conducted as a collaboration between the NCGG and the Lion Foundation for Dental Health. The study complied with the Declaration of Helsinki and was approved by the Ethics Review Committee of the NCGG (approval number 1533) and the Lion Foundation for Dental Health (no. LDH202107). Informed consent was obtained from all study participants and their families prior to study participation. The study is registered in the UMIN Clinical Trials Registry (UMIN000048126).

### Participants

Study participants who attended their first visit to the NCGG outpatient memory clinic from August 2021 to April 2022 and who met the participant selection criteria were informed about the Pearl study orally and in writing. Study participants who agreed to participate in the study were selected.

### Participant exclusion criteria

The exclusion criteria were as follows: 1) A history of brain tumor, subdural hematoma, or head injury resulting in permanent disability; 2) Any focal lesions (identified using computerized tomography or magnetic resonance imaging performed prior to enrollment), such as cerebral infarction, which might substantially affect cognitive function; 3) History of major depression, bipolar disorder, schizophrenia, alcoholism or other drug dependence, or a serious or unstable illness; 4) Vitamin B1, vitamin B12, or folic acid deficiency, syphilis, or thyroid dysfunction that is difficult to treat; 5) Study participants for whom the researcher was unable to perform neuropsychological tests (e.g., CDR or MMSE) or oral function tests; 6) Other cases that the investigator judged not suitable for enrollment.

### Cognitive function assessment and general examination findings

Study participants were classified according to CDR score and using the Peterson criteria [17]. A CDR score of 0 was classified as cognitively normal, and a CDR score of 0.5 as indicating MCI. Dementia was defined as either (1) a CDR score of ≥ 1 or (2) both an MMSE score of ≤ 23 and a CDR score of ≥ 0.5. A study by Saji et al. [16] was used as a reference for CDR diagnosis.

### Potential risk factors and covariates

Data on the following potential risk factors and covariates were collected from the electronic medical records of the NCGG memory clinic: age, sex, smoking status, medication status (number of medications), years of education, body mass index (BMI), and the presence of systemic diseases such as diabetes mellitus, hypertension, dyslipidemia, cardiovascular diseases, cerebrovascular diseases, and mental disorders. These factors were categorized as follows: age (years); BMI (kg/m^2^); years of education (years); sex (male = 0, female = 1); smoking status (no smoking history = 0, smoking history = 1); presence of specific systemic disease (no = 0, yes = 1).

### Assessment of oral function

Number of present teeth, Eichner classification [18], and denture use were examined prior to the oral function tests. The Eichner classification describes the number of occlusal support areas (premolar and molar), as follows: A1–A3: Presence of all four occlusal support areas; B1–B3: Presence of one to three occlusal support areas; B4–C3: No occlusal support areas [18]. The bite force and tongue pressure measurements were performed by one well-trained dentist (Y.I.), and the Repetitive Saliva Swallowing Test (RSST) and oral diadochokinesis (ODK) assessments were performed by six dental hygienists (S.Y., M.M., K.H., M.I., K.N., and M.S.).

For the RSST administration, the dysphagia seminar text book from the Society of Swallowing and Dysphagia of Japan was used to instruct examiners on the correct posture during measurement. The criteria for valid counts and the kappa value of the examiner inter-inspector error was 0.561.

#### Occlusal force

Occlusal force was assessed using a commercial occlusal film according to the manufacturer’s guideline (Dental Prescale II, GC Corporation, Tokyo, Japan) and an analyzer (Bite Force Analyzer, GC Corporation, Tokyo, Japan). The occlusal force of the entire dentition was measured during 3 s of clenching in the maximal intercuspal position. A force < 350 N for the Prescale II (with pressure filter) was diagnosed as reduced occlusal force, following a previous study [19].

#### Oral diadochokinesis (ODK)

ODK was assessed as the number of repetitions for the monosyllables /pa/, /ta/, and /ka/ to evaluate the function of the lips, the tip of the tongue, and the posterior region of the tongue using an automatic counter (Kenkokun Handy, Takei Scientific Instruments Co., Niigata, Japan) [20]. Participants were asked to repeat /pa///ta///ka/ for 5 s each, and the number of times each syllable was pronounced per second was measured. A frequency of < 6 times/s for each of /pa///ta///ka/ was considered to be a reduction in lingual-lip and tongue motor function [21].

#### Repetitive Saliva Swallowing Test (RSST)

The RSST is a simple screening test for dysphagia. The frequency of repetitive salivary swallowing over a 30 s period was measured by examiner finger contact between the laryngeal ridge and the hyoid bone. Six examiners underwent interoperator calibration before the study was performed. A frequency of < 3 swallows was defined as poor swallowing function [22].

#### Tongue pressure

Maximum tongue pressure was measured using a JMS tongue pressure measurement device (TPM-01, JMS Co. Ltd., Hiroshima, Japan). A balloon-type probe was placed on the anterior part of the palate, and participants were asked to press their tongue against their palate with maximum force for several seconds. The pressure was measured three times and the average value was evaluated. A maximum tongue pressure of < 30 kPa was defined as low tongue pressure [21].

### Statistical analysis

Categorical variables are expressed as frequency (*n*) and percentage, and continuous variables are expressed as mean median and standard deviation. The chi-square test or the Kruskal–Wallis test was used to evaluate differences in the potential risk factors among the three groups of participants: cognitively normal, MCI, and dementia. Multinomial logistic regression with backward-forward stepwise method, adjusted for age and BMI was used to clarify the potential risk factors of participants with MCI and dementia compared with those of Cognitively normal participants. Potential risk factors comprised sex, smoking status, number of any drugs used, years of education, hypertension, diabetes mellitus, dyslipidemia, stroke, number of teeth present, occlusal force, ODK score, RSST score, tongue pressure, and Eichner classification. Cardiovascular diseases and mental disease were excluded because they are associated with a wide variety of disease types. Adjusted odds ratios (ORs) and 95% confidence intervals (CIs) were calculated from the multinomial logistic model. All comparisons were two-sided, and *P*-values < 0.05 were considered statistically significant. The data were analyzed using IBM SPSS, version 24.0 (IBM Corp., Armonk, NY, USA).

## Results

**Fig. 1:**
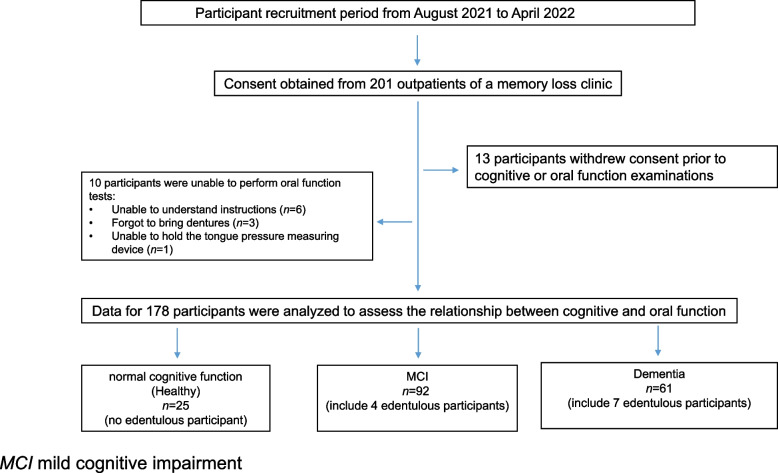
Flowchart of study participants

A total of 201 participants were enrolled in this study. Of these, 13 participants withdrew their consent and 6 participants were unable to understand our explanation and were unable to perform the all oral function tests (Fig. 1). Three participants did not bring their dentures and were unable to bite the occlusal sheet for the occlusal force test. One participant was unable to hold the balloon for measuring tongue pressure on the palate. Finally, 178 participants were classified according to the CDR: 25, 92, and 61 were classified as Cognitively normal, having MCI, and having dementia, respectively (Fig. 1). Normality of age data was assessed using the Shapiro–Wilk test. A normal Q-Q plot is shown in the Additional File.

Table 1 shows the background characteristics of the 178 participants, categorized as cognitively normal (*n* = 25), MCI (*n* = 92), and dementia (*n* = 61) For the dementia group, participants were further stratified based on their CDR scores, with 22 participants having a score of 0.5, 33 participants scoring 1.0, and 6 scoring 2.0. The median MMSE score for the 22 participants with a CDR score of 0.5 was 18, ranging from 13 to 22. Notably, participants with a CDR score of 2.0 were able to perform the oral function tests without significant difficulty, suggesting that this severity level did not impair their testing ability. The median age of Cognitively normal participants was 77.00 years, the sex ratio was 48.0% female, and the mean BMI was 24.1 ± 2.8 kg/m^2^. For participants with MCI, the median age was 79.00 years, the sex ratio was 51.1% female, and the mean BMI was 22.9 ± 3.4 kg/m^2^. For participants with dementia, the median age was 79.00 years, the sex ratio was 49.2% female, and the mean BMI was 22.4 ± 3.3 kg/m^2^. Table 2 compares the number of teeth, dentition, and oral function of Cognitively normal, MCI, and dementia participants. The number of present teeth was higher in cognitively normal participants (21.4 ± 6.6) and MCI participants (20.1 ± 7.8) than in those with dementia (17.0 ± 10.0). However, this difference did not reach statistical significance (*P* = 0.167). The percentage of participants with Eichner classifications of A1 to B3 (i.e., the presence of more than one molar contact) was higher in MCI and Cognitively normal participants (37% to 44%) than in those with dementia (24.6% to 37.7%). The percentage of participants with Eichner classifications of B4 to C3 (i.e., no molar contact) was higher in participants with dementia (37.7%) than in Cognitively normal and MCI participants (16% and 23.9%, respectively).

**Table 1:** Comparisons of background characteristics of Cognitively normal, MCI, and dementia participants

	Cognitively normal (*n* = 25)	MCI (*n* = 92)	Dementia (*n* = 61)	*P*-value
Age (years), median	77.0	79.0	79.0	0.178
Sex, female, *n* (%)	12 (48.0)	47 (51.1)	30 (49.2)	0.951
Smoking, *n* (%)	10 (40.0)	34 (37.0)	23 (37.7)	0.962
No. of drugs taken (*n*), mean ± SD	4.1 ± 3.1	3.3. ± 2.5	4.1 ± 2.7	0.146
Education (years), mean ± SD	12.1 ± 2.7	12.1 ± 2.8	11.0 ± 2.6	0.038
BMI (kg/m^2^), mean ± SD	24.1 ± 2.8	22.9 ± 3.4	22.4 ± 3.3	0.053
CRP (mg/dl), mean ± SD	0.5 ± 1.1	0.2 ± 0.4	0.1 ± 0.2	0.045
HbA1c (%), mean ± SD	5.9 ± 0.6	6.0 ± 0.7	6.2 ± 1.2	0.542
Hypertension, *n* (%)	13 (52.0)	43 (46.7)	36 (59.0)	0.330
Diabetes mellitus, *n* (%)	3 (12.0)	14 (15.2)	10 (16.4)	0.875
Dyslipidemia, *n* (%)	8 (32.0)	34 (37.0)	30 (49.2)	0.208
CVD, *n* (%)	15 (60.0)	23 (25.0)	13 (21.3)	< 0.001
Stroke, *n* (%)	1 (4.0)	15 (16.3)	12 (21.3)	0.143
Mental disease, *n* (%)	0 (0.0)	6 (6.5)	5 (8.2)	0.351

*BMI* Body mass index, *CRP* C-reactive protein, *CVD* Cardiovascular diseases, *HbA1c* Hemoglobin A1c, *MCI* Mild cognitive impairment, *SD* Standard deviation

**Table 2:** Comparison of number of teeth, Eichner classification, and oral function in Cognitively normal, MCI, and dementia participants

	Cognitively normal (*n* = 25)	MCI (*n* = 92)	Dementia (*n* = 61)	*P*-value
No. of present teeth, mean ± SD	21.4 ± 6.6	20.1 ± 7.8	17.0 ± 10.0	0.167
Edentulous participants, *n* (%)	0 (0)	4 (4.3)	7 (11.4)	N/A
Denture use, *n* (%)	14 (56.0)	44 (48.0)	29 (48.0)	0.744
Eichner classification^a^				
A1-A3, *n* (%)	11 (44.0)	34 (37.0)	23 (37.7)	0.144
B1-B3, *n* (%)	10 (40.0)	36 (39.1)	15 (24.6)	
B4-C3, *n* (%)	4 (16/0)	22 (23.9)	23 (37.7)	
Occlusal force ≥ 350 N, *n* (%)	19 (76.0)	51 (55.4)	27 (44.3)	0.026
Oral diadochokinesis^b^				
/pa/	18 (72.0)	39 (42.3)	17 (27.9)	< 0.001
/ta/	14 (56.0)	36 (39.1)	15 (24.6)	0.017
/ka/	13 (52.0)	21 (22.8)	9 (14.6)	0.001
RSST score, *n* (%)^c^	21 (84.0)	63 (68.5)	34 (54.8)	0.034
Tongue pressure ≥ 30 kPa, *n* (%)	13 (52.0)	36 (39.1)	19 (31.1)	0.189

*MCI* mild cognitive impairment, *RSST* Repetitive Saliva Swallowing Test, *SD* standard deviation

^a^Eichner classification [18]: According to the remaining status of the four occlusal support areas of the upper and lower jaws by the left and right molar groups. A1-A3: State with all four occlusal support areas. B1-B3: State with one to three occlusal support areas. B4-C3: State without occlusal support area

^b^Oral diadochokinesis: Number of participants who could pronounce six syllables per second

^c^RSST: Number of participants who could swallow at least three times in 30 s

Table 3 shows the crude ORs and 95% CIs and Table 4 shows the adjusted ORs and 95% CIs from the multinomial logistic regression model of the onset of MCI or dementia. The absence of multicollinearity of the independent variables was checked before conducting the multinomial logistic regression analysis. BMI was associated with a high risk of the onset of MCI or dementia, with an adjusted OR of 0.773 for participants with MCI (95% CI: 0.626–0.954, *P* = 0.017) and 0.683 for those with dementia (95% CI: 0.546–0.854, *P* = 0.001). ODK /ka/ was significantly associated with the onset of MCI and dementia, but the wide CIs for an adjusted OR of 6.930 for participants with MCI (95% CI: 1.284–37.402, *P* = 0.024) and 7.382 for those with dementia (95% CI: 1.068–51.006, *P* = 0.043) indicate substantial uncertainty about the estimate. This suggests that the results may have been affected by sample size limitations and other potential confounding factors such as lifestyle factors and socioeconomic status. RSST score showed a tendency towards an association with a high risk of dementia onset, with an adjusted OR of 4.171 for participants with dementia (95% CI: 0.981–17.736, *P* = 0.053). Although this result approached statistical significance, the wide CI indicates a considerable level of uncertainty about the precision of this estimate. The absence of dyslipidemia was associated with a low risk of dementia onset, with an adjusted OR of 0.209 95% CI: 0.057–0.770, *P* = 0.019). The absence of stroke was associated with a low risk of dementia onset, with an adjusted OR of 0.103 (95% CI: 0.010–1.074, *P* = 0.057). Other factors, such as sex, smoking status, number of drugs taken, education years, number of present teeth, hypertension, diabetes mellitus, ODK/pa/ and /ta/, tongue pressure, occlusal force, and Eichner classification were not associated with a risk of MCI or dementia (Table 4).

**Table 3:** Crude multinomial logistic regression results for the onset of MCI or dementia

	OR	95% CI	*P*-value
MCI			
Age	1.044	0.956–1.141	0.340
Sex	0.553	0.151–2.026	0.371
Smoking status	0.684	0.177–2.645	0.582
BMI^a^	0.773	0.626–0.954	0.017*
No. of drugs taken	0.824	0.657–1.033	0.093
Education years	1.010	0.833–1.224	0.921
No. of present teeth	0.992	0.849–1.159	0.921
Hypertension	1.719	0.537–5.498	0.361
Dyslipidemia	0.463	0.139–1.540	0.209
Diabetes	0.756	0.128–4.480	0.758
Stroke	0.135	0.014–1.297	0.083
ODK/pa/	2.526	0.638–9.998	0.187
ODK/ta/	0.507	0.104–2.468	0.400
ODK/ka/	6.533	1.185–36.010	0.031*
RSST score	2.348	0.596–9.245	0.222
Tongue pressure ≥ 30 kPa	0.500	0.136–1.844	0.298
Occlusal force ≥ 350 N	2.390	0.554–10.317	0.243
Eichner classification			
C1–C3	0.945	0.039–22.888	0.972
B1–B3	0.650	0.158–3.157	0.650
Dementia			
Age	1.044	0.945–1.152	0.399
Sex	0.654	0.154–2.773	0.565
Smoking status	0.538	0.119–2.425	0.420
BMI^a^	0.683	0.546–0.854	0.001*
No. of drugs taken	0.907	0.707–1.164	0.444
Education years	0.850	0.676–1.069	0.165
No. of present teeth	0.902	0.767–1.061	0.214
Hypertension	1.476	0.407–5.353	0.554
Dyslipidemia	0.204	0.054–0.763	0.018*
Diabetes	1.191	0.182–7.791	0.855
Stroke	0.103	0.010–1.074	0.057
ODK/pa/	3.225	0.706–14.735	0.131
ODK/ta/	0.619	0.101–3.796	0.604
ODK/ka/	6.911	0.978–48.848	0.053
RSST score	4.242	0.993–18.123	0.051
Tongue pressure ≥ 30 kPa	0.546	0.133–2.231	0.399
Occlusal force ≥ 350 N	1.802	0.372–8.735	0.464
Eichner classification			
C1–C3	0.295	0.010–8.558	0.477
B1–B3	0.304	0.056–1.638	0.166

*BMI* Body mass index, *CI* Confidence interval, *MCI* Mild cognitive impairment, *ODK* Oral diadochokinesis, *OR* Odds ratio, *RSST* Repetitive Saliva Swallowing Test

^a^BMI, age, education years, no. of present teeth, and no. of drugs taken were continuous variables. The remaining independent variables were nominal variables (male = 0, female = 1; no smoking history = 0, smoking history = 1; no hypertension = 0, hypertension = 1; no dyslipidemia = 0, dyslipidemia = 1; no stroke = 0, stroke = 1; possibility of ODK = 1, no possibility of ODK = 0; possibility of RSST score = 1, no possibility of RSST score = 0; possibility of tongue pressure ≥ 30 kPa = 1, no possibility of tongue pressure ≥ 30 kPa = 0; possibility of occlusal force ≥ 350 N = 1, no possibility of occlusal force ≥ 350 N = 0; Eichner classification C1–C3 = 0, B1–B2 = 1, A1–A2 = 2)

^*^P < 0.05, logistic regression analysis

**Table 4:** Adjusted multinomial logistic regression results for the onset of MCI or dementia

	OR	95% CI	*P*-value
MCI			
Sex	0.664	0.192–2.297	0.518
Smoking status	0.881	0.254–3.060	0.842
BMI^a^	0.781	0.635–0.961	0.019*
No. of drugs taken	0.845	0.680–1.050	0.129
Education years	1.000	0.828–1.208	0.999
No. of present teeth	0.987	0.847–1.150	0.866
Hypertension	1.811	0.574–5.716	0.311
Dyslipidemia	0.474	0.145–1.545	0.216
Diabetes	0.859	0.150–4.918	0.864
Stroke	0.139	0.015–1.299	0.083
ODK/pa/	2.687	0.674–10.710	0.161
ODK/ta/	0.493	0.100–2.425	0.385
ODK/ka/	6.930	1.284–37.402	0.024*
RSST score	2.310	0.589–9.059	0.230
Tongue pressure ≥ 30 kPa	0.571	0.164–1.982	0.377
Occlusal force ≥ 350 N	2.311	0.544–9.820	0.256
Eichner classification			
C1–C3	1.098	0.049–24.838	0.953
B1–B3	0.771	0.174–3.409	0.732
Dementia			
Sex	0.774	0.190–3.142	0.720
Smoking status	0.684	0.166–2.829	0.600
BMI^a^	0.683	0.546–0.854	0.001*
No. of drugs taken	0.907	0.707–1.164	0.444
Education years	0.842	0.672–1.055	0.135
No. of present teeth	0.898	0.765–1.054	0.188
Hypertension	1.560	0.437–5.573	0.494
Dyslipidemia	0.209	0.057–0.770	0.019*
Diabetes	1.353	0.213–8.574	0.749
Stroke	0.105	0.010–1.074	0.057
ODK/pa/	3.424	0.744–15.764	0.114
ODK/ta/	0.604	0.098–3.725	0.587
ODK/ka/	7.382	1.068–51.006	0.043*
RSST score	4.171	0.981–17.736	0.053
Tongue pressure ≥ 30 kPa	0.614	0.158–2.386	0.481
Occlusal force ≥ 350 N	1.740	0.363–8.334	0.488
Eichner classification			
C1–C3	0.345	0.013–9.397	0.528
B1–B3	0.331	0.062–1.770	0.196

*BMI* Body mass index, *CI* Confidence interval, *MCI* Mild cognitive impairment, *ODK* Oral diadochokinesis, *OR* Odds ratio, *RSST* Repetitive Saliva Swallowing Test

^a^BMI, education years, no. of present teeth, and no. of drugs taken were continuous variables. The remaining independent variables were nominal variables (male = 0, female = 1; no smoking history = 0, smoking history = 1; no hypertension = 0, hypertension = 1; no dyslipidemia = 0, dyslipidemia = 1; no stroke = 0, stroke = 1; possibility of ODK = 1, no possibility of ODK = 0; possibility of RSST score = 1, no possibility of RSST score = 0; possibility of tongue pressure ≥ 30 kPa = 1, no possibility of tongue pressure ≥ 30 kPa = 0; possibility of occlusal force ≥ 350 N = 1, no possibility of occlusal force ≥ 350 N = 0; Eichner classification C1–C3 = 0, B1–B2 = 1, A1–A2 = 2)

^*^P < 0.05, logistic regression analysis

## Discussion

In this study, we investigated the relationship between cognitive decline and oral function in memory clinic outpatients. Although the number of teeth present was higher in cognitively normal participants (21.4 ± 6.6) and MCI participants (20.1 ± 7.8) than in those with dementia (17.0 ± 10.0), no significant relationship was observed *(P* = 0.167) between the number of present teeth and cognitive decline in this study. However, BMI and ODK were significantly associated with the development of MCI and dementia, and RSST score showed no association with MCI and a trend for an association with dementia. These findings raise the possibility that, even if patients retain masticatory function, poor tongue movement may contribute to both impaired nutritional intake and reduced cognitive function. The present findings may provide useful insights for future research into the potential causal relationship between poor tongue movement and cognitive decline.

### BMI and cognitive decline

BMI, which was included in the model as a covariate, was significantly associated with MCI and dementia. Previous studies have shown that being obese at < 65 years of age and being underweight at ≥ 65 years is a risk for dementia [23]. Kivimäki et al. used stepwise stratification to compare the risk of developing dementia by progressively classifying the timing of BMI assessment and age at dementia onset over a follow-up period. They found that higher BMI was associated with an increased risk of dementia when BMI was assessed more than 20 years before dementia diagnosis, but lower BMI was associated with increased dementia risk when BMI was assessed less than 10 years after dementia diagnosis [24]. Given these findings, the fact that higher BMI was significantly associated with lower risk of dementia and MCI in the present study may be because the average participant age was over 75 years, and because BMI was assessed at the same time as cognitive function diagnosis.

As dysphagia has been previously reported to be associated with lower BMI [25], and MCI and dementia were strongly associated with BMI in the present study, further research is needed to determine whether dysphagia is related to cognitive decline via lower BMI.

### Tooth loss and cognitive decline

Older individuals with edentulous jaws or no functional dentition are at higher risk of malnutrition [26], and tooth loss has been shown to be associated with cognitive impairment and increased risk of dementia [27, 28]. Experimental extraction of maxillary molars from aged mice showed a reduction in *Bdnf* gene expression; brain-derived neurotrophic factor feeds cerebral nerves in the hypothalamus and hippocampus [29]. In the present study, no association was found between the number of present teeth and cognitive decline in participants with MCI and dementia, but previous studies have reported an association [30]. Therefore, it is difficult to conclude that there is no association between the number of present teeth and cognitive function without also evaluating denture suitability, masticatory ability, and other factors.

### RSST and cognitive decline

During the oral phase of normal swallowing, food is chewed and mixed with saliva to move the food to the back of the mouth. In people with dementia, xerostomia and tongue weakness affects prolonged chewing of food residues in the mouth [31]. Central dysphagia is caused by neurological disorders such as cerebrovascular disease (cerebral infarction, cerebral hemorrhage, subarachnoid hemorrhage), Parkinson’s disease, Alzheimer’s disease, and amyotrophic lateral sclerosis [32–34]. The present study utilized the RSST, a simple screening tool for dysphagia. However, the RSST is considered less accurate than more advanced diagnostic methods, such as the video fluoroscopic swallow test, neck auscultation, and the water swallow test, for assessing dysphagia [35–37]. In this study, no significant association was found between MCI and the RSST score (OR: 2.348, 95% CI: 0.596–9.245, *P* = 0.222). However, a trend suggesting an association with dementia was observed (OR: 4.171, 95% CI: 0.981–17.736, *P* = 0.053).

While RSST score may not be a reliable indicator of cognitive decline in the early stages, it could provide valuable insights as the severity of cognitive impairment increases. Nevertheless, these results warrant further validation owing to the wide CIs, which introduce substantial uncertainty about the estimated ORs.

### ODK and cognitive decline

ODK is reduced in individuals with cognitive decline and Parkinson’s disease [38]. Watanabe et al. reported that ODK/pa/ was strongly associated with presence of MCI but tooth number and occlusal force were not [39]. The different results for ODK/pa/ in the present study may be because the mean number of ODK/pa/ pronunciations was lower than in the previous study [39] (Tables 3, 4), participants were an average of more than 6 years older, and ODK was analyzed as a nominal variable. ODK/ta/ has been found to be associated with lower tongue pressure [40], perhaps because the syllable pronunciation of /ta/ is similar to the pressure contact of the tongue apex to the palate during the tongue pressure measurement. Moreover, the two previous studies were conducted on community residents, and the small number of Cognitively normal elderly participants in the present study may have affected the results.

Dysphagia is associated with abnormal hyoid bone position [41, 42] and prolonged pharyngeal swallow reflex time [42, 43]. The glossopharyngeal and vagus nerves control the pharyngeal phase swallowing reflex [44, 45]. The glossopharyngeal nerve controls sensations at the tongue root and pharynx, and the hypoglossal nerve controls tongue movement [46]. As the pronunciation of ODK/ka/, which was found to be relevant in the present study, requires elevation of the root of the tongue as during swallowing, the harmonic activity of the hypoglossal, glossopharyngeal, and vagus nerves is closely related to both swallowing and ODK/ka/ pronunciation. Ogino et al. also reported that ODK/ka/ pronunciation is associated with swallowing function in elderly people living in the community [47]. Although both ODK/ka/ and RSST performance require a similar movement of the tongue root elevation, the lack of association between RSST and cognitive function in this study may be because of the small number of participants (particularly Cognitively normal participants). The use of a more quantitative assessment of swallowing function may have detected an association. Therefore, it seems reasonable that ODK/ka/score is associated with functional decline as cognitive function declines. Additional research is needed to determine whether ODK and other oral function assessments in dentistry can be used to screen for cognitive decline.

### Study limitations

There were several study limitations. Notably, the wide CIs around the ORs for ODK /ka/ indicate substantial uncertainty about the findings, probably partly because of the limited sample size. Additional research using larger samples is needed to further clarify this association.

Furthermore, we did not calculate the optimal sample size; selection bias may have affected the results because the number of Cognitively normal participants was less than half the number of those with MCI or dementia. The participants in this study were outpatients visiting an outpatient memory clinic, so the caution is needed before generalizing the findings, as these study participants were more likely than the local population to have subjective and objective symptoms of cognitive decline. The CDR cognitive function assessment was based primarily on interviews with the individuals and family members. The disease type of some participants in the dementia group could not be classified, and because of the small sample, the number of cases was insufficient to conduct comparisons by disease type.

Another limitation is the potential for examiner bias or inconsistencies in the measurement process, as oral function tests were performed by a single well-trained dentist. Single examiner errors can arise from inconsistencies in measurement techniques, unconscious bias, or subjective expectations influencing the outcomes. These errors can affect the reliability and validity of the results, especially in studies where precise and objective measurements are essential. Additional studies using multiple examiners or objective measurement tools would be useful to increase the robustness of the findings.

The possibility of reverse causality should also be considered. Because of the cross-sectional study design, the direction of causality in the observed association is not clear. It is possible that cognitive decline may occur first and oral function may decline as a result. To clarify the direction of this association, studies using longitudinal designs are needed to examine temporal causality in more detail.

### Future prospects

One study showed that an oral hygiene instruction and oral function training MA2participants with a ≥ 4 mm probing depth, tongue pressure, and ODK/ta/ in older participants with MMSE scores between 21 and 26 [48]. Therefore, we believe that a well-designed cohort intervention study that records the timing of tooth extraction in participants could clarify the relationship between causal cognitive decline and swallowing and ODK. In Japan, there are approximately 68,000 dental clinics, far outnumbering the 3,000 dementia outpatient clinics. This makes dental clinics more accessible to individuals of all ages, particularly in rural areas. The results of this study suggest that dentists could play a key role in identifying older individuals at risk of reduced ODK function, potentially offering an opportunity for early intervention. Collaboration between dental and medical professionals may improve early diagnosis and treatment of cognitive decline, providing an avenue for proactive care and management in at-risk populations.

## Conclusion

Although ODK /ka/ scores were found to be significantly associated with both dementia and MCI, the wide CIs of the adjusted ORs indicate substantial uncertainty in these estimates. This suggests that the observed associations may be affected by the limited sample size and other potential confounding factors. Therefore, although the findings suggest that ODK /ka/ may be a useful marker for identifying individuals with possible MCI, additional studies with larger sample sizes and robust statistical controls are needed to confirm these results and to increase their reliability. Such studies could provide more definitive evidence on the potential of ODK /ka/ as a screening tool for cognitive decline.

## Supplementary Information

Supplementary Material 1.

## Data Availability

Data availability The datasets generated and analyzed during the current study are available from the corresponding author upon reasonable request.

## Abbreviations

MMSE: Mini-Mental State Examination
CDR: Clinical Dementia Rating
MCI: Mild cognitive impairment
NCGG: National Center for Geriatrics and Gerontology
ODK: Oral diadochokinesis
RSST: Repetitive Saliva Swallowing Test
BMI: Body mass index
ORs: Odds ratios
CIs: Confidence intervals
