# Supplementary material for: Oral diadochokinesis performance correlates with mild cognitive impairment: a cross-sectional study
Source: BMC Oral Health. 2025 Jun 3;25:891. doi: 10.1186/s12903-025-06289-4 (PMC12135609; doi:10.1186/s12903-025-06289-4)
Supplement: Supplementary file 1 — Supplementary Material 1. [file 12903_2025_6289_MOESM1_ESM.pptx]

## Slide 1
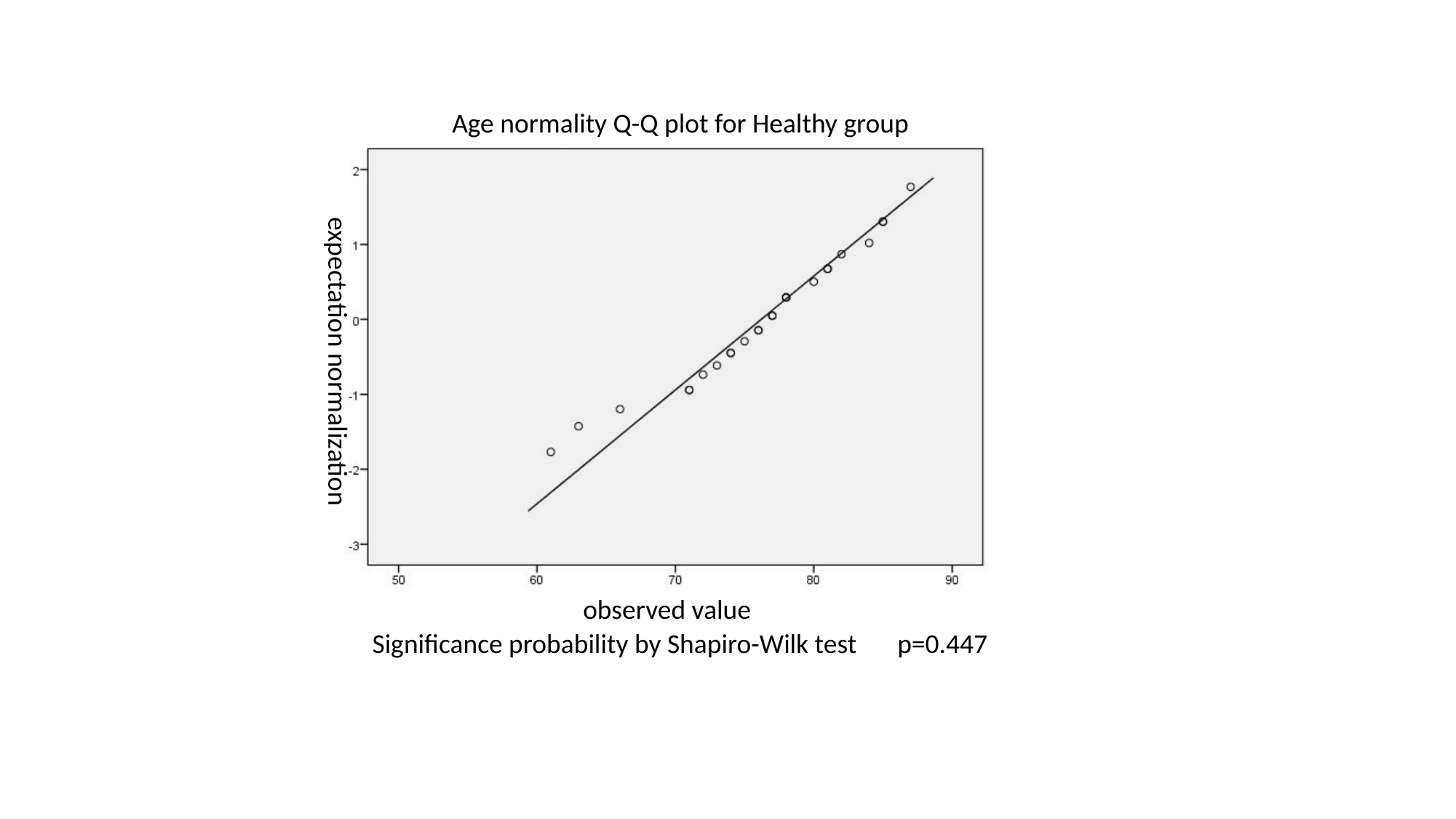

Age normality Q-Q plot for Healthy group
expectation normalization
observed value
Significance probability by Shapiro-Wilk test　p=0.447

## Slide 2
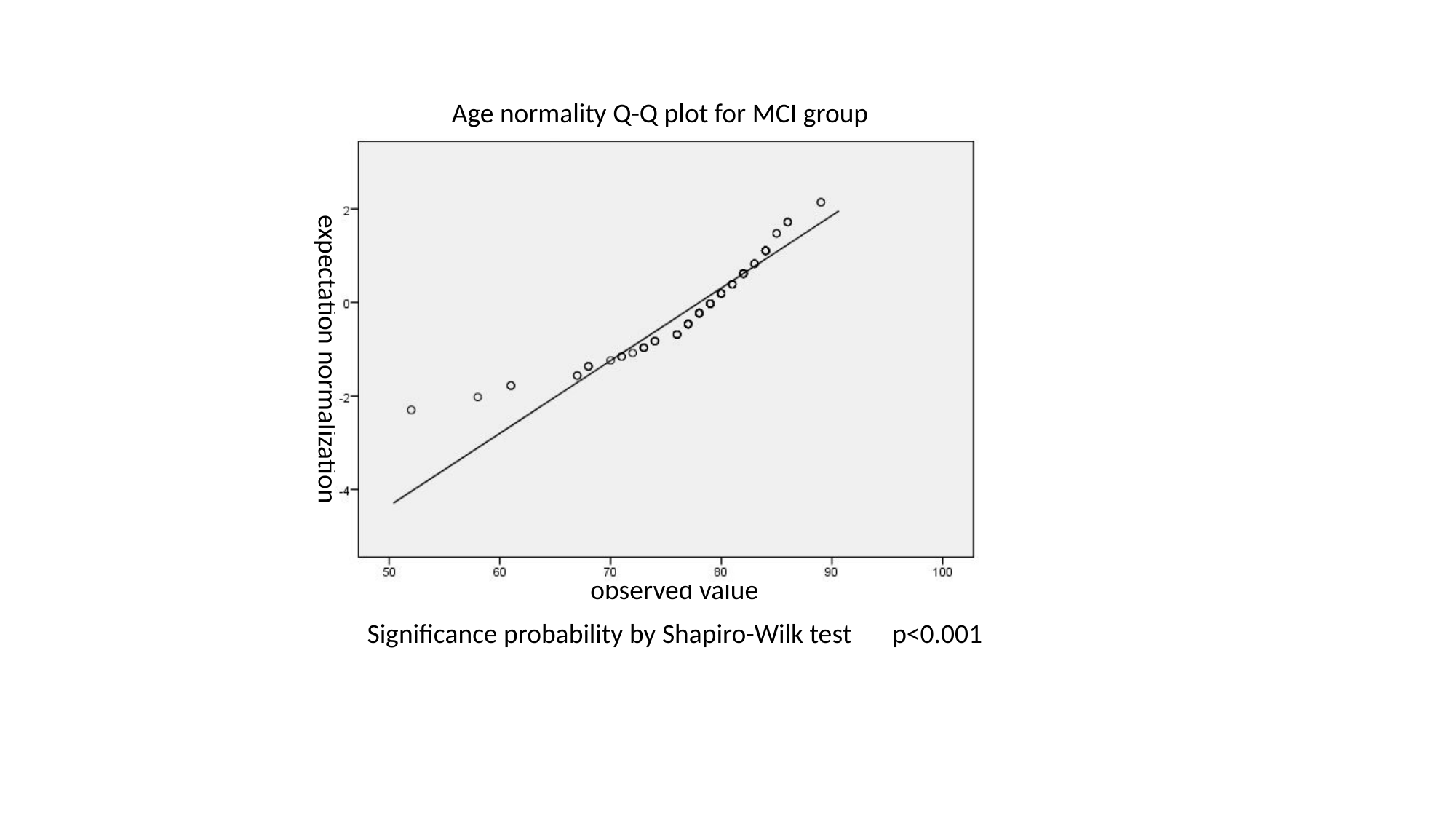

Age normality Q-Q plot for MCI group
expectation normalization
observed value
Significance probability by Shapiro-Wilk test　p<0.001

## Slide 3
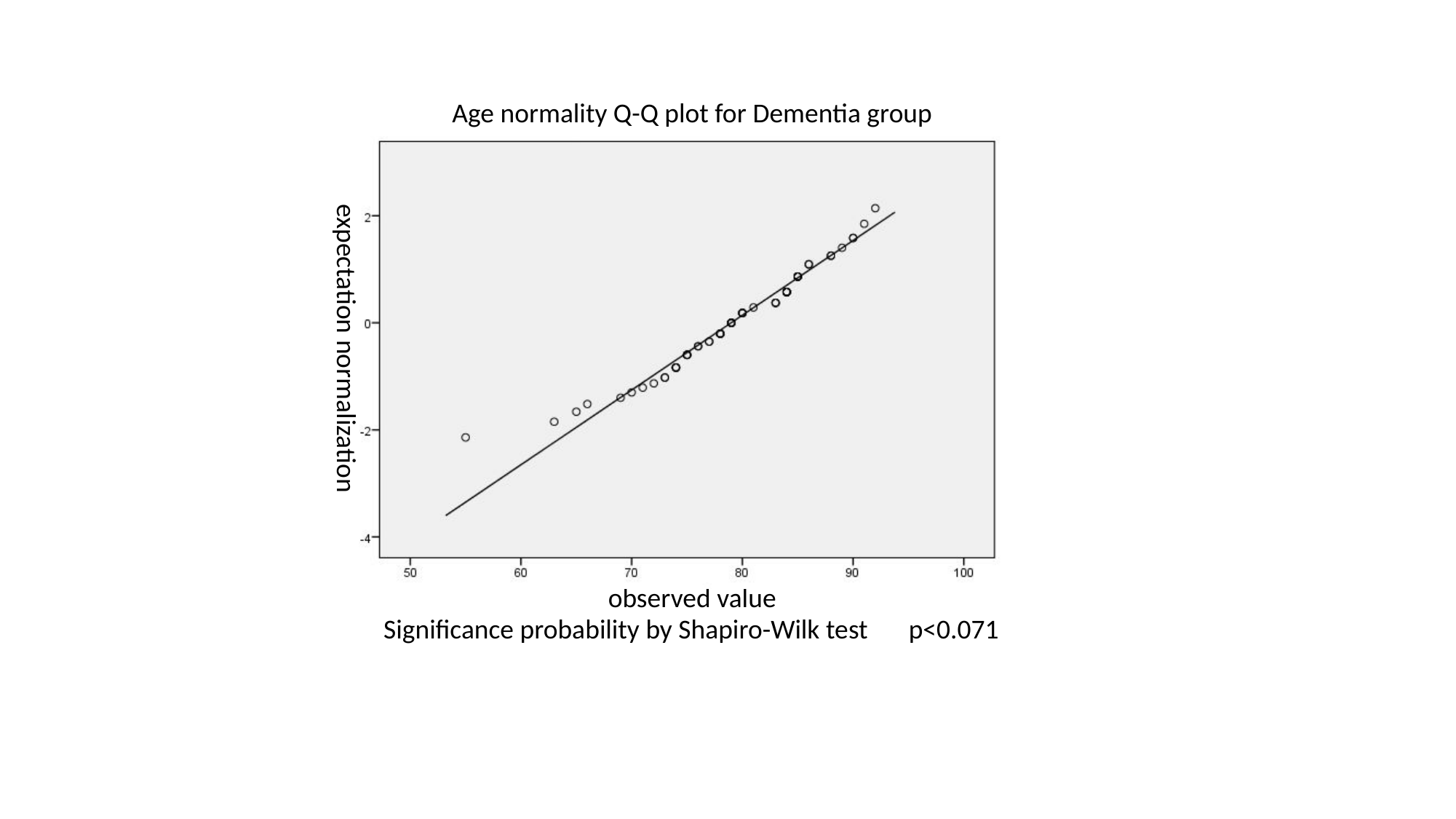

Age normality Q-Q plot for Dementia group
expectation normalization
observed value
Significance probability by Shapiro-Wilk test　p<0.071
